# Supplementary material for: The Parkinson's disease related mutant VPS35 (D620N) amplifies the LRRK2 response to endolysosomal stress
Source: Biochem J. 2024 Feb 15;481(4):265–78. doi: 10.1042/BCJ20230492 (PMC10903469; doi:10.1042/BCJ20230492)
Supplement: Supplementary Material [file BCJ-481-265-s1.pdf]

Supplementary Figure Legends

Figure S1: LLOMe, nigericin and chloroquine cause distinct responses to endolysosomal stress

A. Representative western blot of RPE1 Flp-In Parental cells pre-treated with Vehicle (DMSO), 150 nM apilimod (Apil), 100 nM concanamycin A (ConA) or 100 nm MLi-2 then treated with Vehicle (DMSO), 500 μM LLOMe or 2 μM nigericin for 2 h prior to lysis.

B. Quantification of A. Values normalised to vehicle control. n = 2. Error bars indicate mean and range.

C. Schematic of mKeima-Gal3 (lyso-Keima) reporter principle

D. Representative images of RPE1 Flp-In HA-VPS35 WT cells (not induced with doxycycline) expressing mKeima-Gal3 treated for 1 h with LLOMe, nigericin or chloroquine (CQ) and then imaged immediately or imaged following a 12 h washout. Scale bar 10 μm.

E. Representative images of RPE1 Flp-In Parental cells treated with LLOMe or vehicle (DMSO) and nigericin or vehicle (EtOH) for the indicated times then fixed and stained for LC3 and LAMP1. Scale bar 10 μm.

F. Representative images of RPE1 Flp-In Parental cells treated with LLOMe or nigericin for 30 min then fixed and stained for markers for the ESCRT machinery (CHMP2B, ALIX) and late endosomes/lysosomes (CD63, LAMP1). Scale bar 10 μm.

Figure S2: The VPS35 (D620N) mutation does not affect ESCRT recruitment in response to lysosomal membrane damage

A. Representative images of doxycycline-induced (24 h) RPE1 Flp-In HA-VPS35 WT and (D620N) cells treated with 500 μM LLOMe for 15 or 30 min prior to fixation and staining with the indicated antibodies. Scale bar 10 μm.

B. Quantification of the number and average size of CHMP2B puncta per cell and the mean CHMP2B intensity within LAMP1 puncta per cell. 37-60 cells quantified per condition, n = 2. One-way ANOVA with Tukey's multiple comparisons test. ns, not significant.

C. Subcellular fractionation of RPE1 Flp-In HA-VPS35 WT and D620N (DN) cells induced with doxycycline for 24 h and then treated with 500 μM LLOMe, 3 μM nigericin or vehicle control (DMSO) for 60 min. PNS; post nuclear supernatant, TPS; total protein stain

D. Quantification of D. Values normalised to WT control. n = 3. Error bars indicate mean ± SD. One-way ANOVA with Tukey's multiple comparisons test. P * < 0.05, P ** < 0.01.

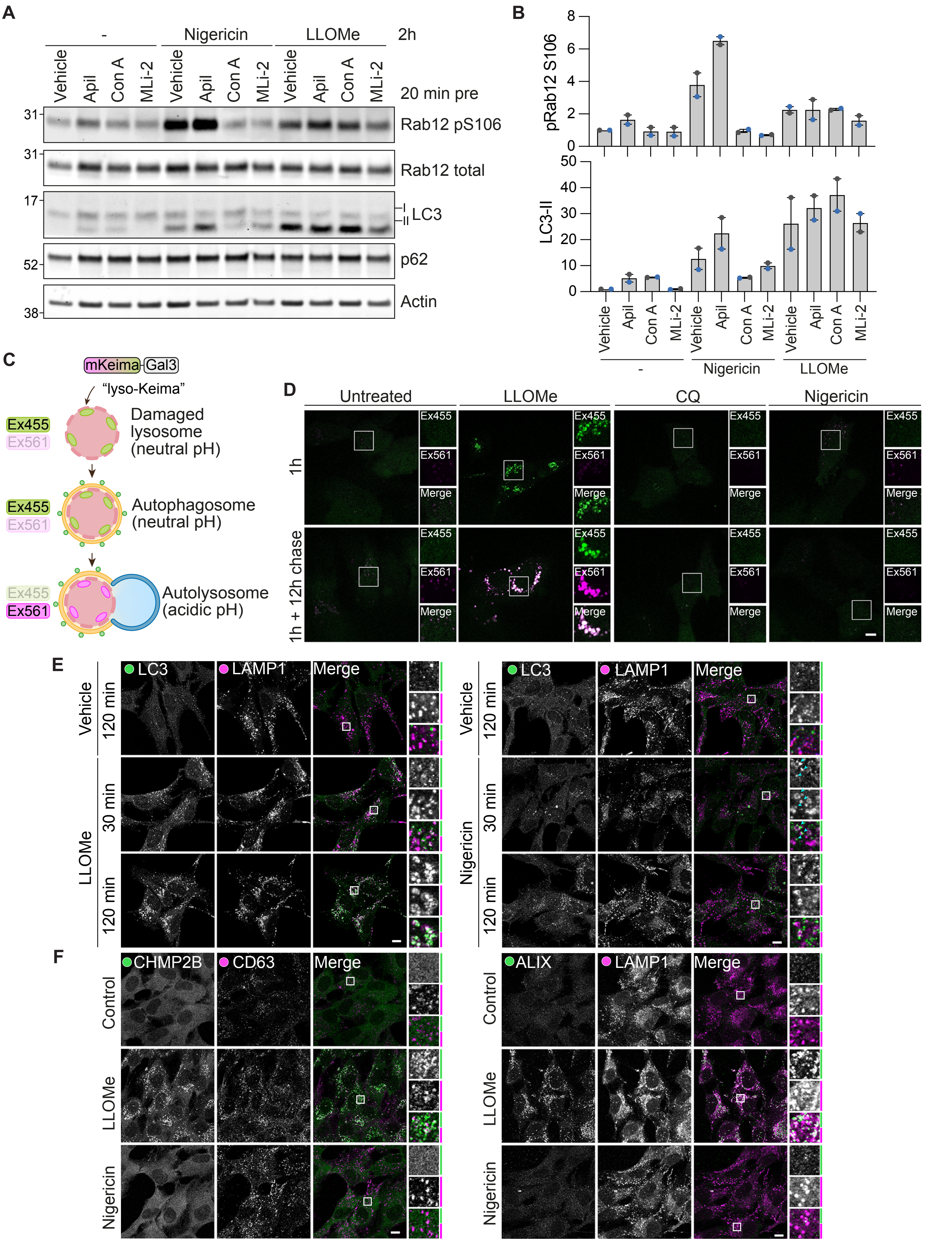
A
-
Nigericin
LLOMe
2h
Vehicle
Apil
Con A
MLi-2
20 min pre
Rab12 pS106
Rab12 total
LC3
I
II
p62
Actin
31
17
52
38
B
pRab12 S106
LC3-II
C
mKeima
Gal3
"lyso-Keima"
Ex455
Ex561
Damaged lysosome (neutral pH)
Autophagosome (neutral pH)
Autolysosome (acidic pH)
D
Untreated
LLOMe
CQ
Nigericin
1h
1h + 12h chase
Merge
E
LC3
LAMP1
ALIX
Vehicle
120 min
30 min
F
CHMP2B
CD63
Control

Figure S1

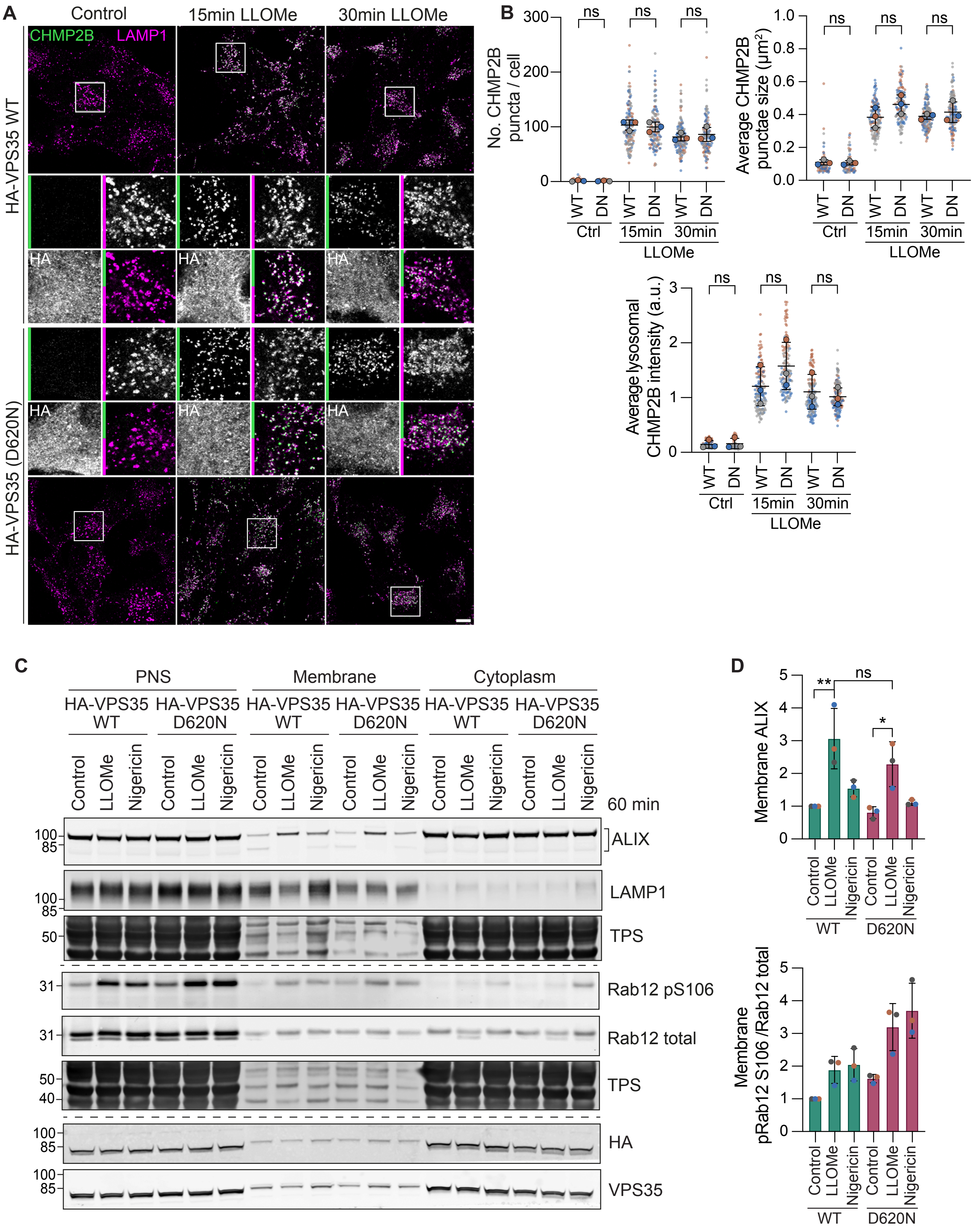

A
Control
15min LLOMe
30min LLOMe
CHMP2B
LAMP1
HA-VPS35 WT
HA-VPS35 (D620N)
HA
B
No. CHMP2B puncta / cell
Average CHMP2B punctae size (µm²)
Average lysosomal CHMP2B intensity (a.u.)
ns
WT
DN
Ctrl
15min
30min
LLOMe
C
PNS
Membrane
Cytoplasm
HA-VPS35 WT
HA-VPS35 D620N
Control
LLOMe
Nigericin
60 min
ALIX
LAMP1
TPS
Rab12 pS106
Rab12 total
HA
VPS35
D
Membrane ALIX
Membrane pRab12 S106 /Rab12 total
WT
D620N

Figure S2

	[D620N] Mutant phenotype previously reported		RPE1 FlpIn VPS35 [D620N] model
	**Effect**	**Reference**	
LRRK2 activation	Enhanced	[22,23]	Enhanced
VPS35 association with WASH complex	Impaired	[14]<br>[15]	Impaired
Endosome localisation of WASH complex	Unaffected	[14]	Unaffected
	Impaired	[15]	
CIM6PR trafficking	Unaffected	[15, 29]	Unaffected
	Impaired	[14, 30-32]	
Sortilin trafficking	Unaffected	[29]	Unaffected

Table 1 - comparison of results obtained with the VPS35 FlpIn cell model developed in this study with previous literature reports, relying on different contexts and configurations.
